# iDLB-Pred: identification of disordered lipid binding residues in protein sequences using convolutional neural network

**DOI:** 10.1038/s41598-024-75700-x

**Published:** 2024-10-21

**Authors:** Sharaf J. Malebary, Nashwan Alromema

**Affiliations:** 1 Department of Information Technology, Faculty of Computing and Information Technology-Rabigh, King Abdulaziz University, P.O. Box 344, 21911 Rabigh, Saudi Arabia; 2 Department of Computer Science, Faculty of Computing and Information Technology-Rabigh, King Abdulaziz University, P.O. Box 344, 21911 Rabigh, Saudi Arabia

**Keywords:** Intrinsic disordered regions, Disordered lipid binding residues, Ten-fold cross validation, Feature description, Convolutional neural network, Protein sequence analyses, Computational biology and bioinformatics, Computational models

## Abstract

Proteins, nucleic acids, and lipids all interact with intrinsically disordered protein areas. Lipid-binding regions are involved in a variety of biological processes as well as a number of human illnesses. The expanding body of experimental evidence for these interactions and the dearth of techniques to anticipate them from the protein sequence serve as driving forces. Although large-scale laboratory techniques are considered to be essential for equipment for studying binding residues, they are time consuming and costly, making it challenging for researchers to predict lipid binding residues. As a result, computational techniques are being looked at as a different strategy to overcome this difficulty. To predict disordered lipid-binding residues (DLBRs), we proposed iDLB-Pred predictor utilizing benchmark dataset to compute feature through extraction techniques to identify relevant patterns and information. Various classification techniques, including deep learning methods such as Convolutional Neural Networks (CNNs), Deep Neural Networks (DNNs), Multilayer Perceptrons (MLPs), Recurrent Neural Networks (RNNs), Long Short-Term Memory (LSTM) networks, and Gated Recurrent Units (GRUs), were employed for model training. The proposed model, iDLB-Pred, was rigorously validated using metrics such as accuracy, sensitivity, specificity, and Matthew’s correlation coefficient. The results demonstrate the predictor’s exceptional performance, achieving accuracy rates of 81% on an independent dataset and 86% in 10-fold cross-validation.

## Introduction

The physicochemical characteristics of the lateral side chain of a DLBR play a crucial role in its ability to bind lipids. IDRs, are parts of protein sequence that do not have a stable three-dimensional structure under physiological conditions. Many proteins contain one or more of these IDRs^1,2^. Current research has shown that proteins containing IDRs are widespread in all spheres of life and are responsible for a wide variety of biological processes^3^. In instance, it was found that IDRs interact with a number of different small molecules, as well as DNA, RNA, proteins, and lipids^4–6^. Yet, the experimental annotation of only a few hundred of these interactions was completed^7^. Because of this gap in the annotation, computational approaches that utilized protein sequences to anticipate IDRs interrelating with certain associate classes have been developed^8^.

Nuclear magnetic resonance spectroscopy (NMR), small-angle X-ray scattering (SAXS), and other bioinformatics tools have been used to examine IDRs. NMR can indicate the population of distinct conformations within the structural ensemble of IDRs^9,10^. SAXS can reveal the overall shape and flexibility of disordered areas in solution. By analyzing sequence composition, disorder predictors, and conservation patterns, bioinformatics techniques may predict the presence and features of IDRs in protein sequences^11^. IDRs are known to interrelate with a diverse set of association, including proteins, nucleic acids, and lipids; however, almost all of the currently available predictors concentrate on the protein-binding IDRs^12^. A current study found 21 models of IDRs that interrelate to proteins. These IDRs were identified using a survey. Methods such as ANCHOR^13,14^MoRFpred^15^, MoRFChiBi^16^, and OPAL^17^ are examples of some of the most well-known algorithms in this category. On the other hand, there is just one approach, called DisoRDPbind^18^, for the forecasting of the IDR’s that interrelate with DNA and RNA, and there are no methods that consider for the linkage with lipids. The absence of techniques might be described by the limited range of exploratory which was required to train and evaluate the performance of models. These models needed to be trained and evaluated before they could be used.

In spite of this, the most recent versions of the DisProt database have incorporated a substantial number of additional experimental annotations. To be more explicit, in comparison to the version 7.2^19^ that came before it, Disprot version 8.0 offers approximately 50% more experimental annotations of the IDRs for lipid binding. It has been shown that lipids play a part in a huge variety of cellular procedures, having the storage of energy, signaling, control, insulation, and transport^20,21^. Their many functions underscore the relevance of lipids in cellular homeostasis and appropriate cell and organismal functioning^22,23^.

The interactions between proteins and lipids are significantly influenced by the intrinsic disorder. According to research, the improper folding of certain IDR-containing proteins might alter the affinity of such proteins for lipids, which can lead to a wide range of disorders^24^. For example, the improper folding of the totally disordered lipid-binding a-synuclein protein as well as the considerably disordered tau protein has been linked to a number of neurodegenerative illnesses^25–28^. Another illustration of an interrelation between IDRs and a lipid bilayer is provided by the SecA protein found in Escherichia coli. In addition, many bacteriocins, such as colicin A, are capable of performing membrane insertion only after they have unfolded into the condition of disorganized bubbling granules. This happens when the bacteriocins interact with the target cell’s cytoplasmic lipids^29^.

We proposed iDLB-Pred, a predictor of disordered lipid-binding residues, encouraged by the current expansion in the discovery of the lipid-interacting IDRs and their physiological significance. iDLB-Pred was developed in response to these factors (DLBRs). DLBRs have an innately disordered structure, have the ability to interact with lipids, and do not have any transmembrane sections. This indicates that the predictions given by iDLB-Pred are a useful addition to the data generated by the currently available predictors of the transmembrane regions. The input protein sequence is fed into iDLB-Pred, which then uses a deep neural network to estimate the probability of lipid adsorption in unstructured areas for each amino acid in the order. The creation of this gadget incorporates a number of different novelties into its design.

## Materials and methods

**Fig. 1:**
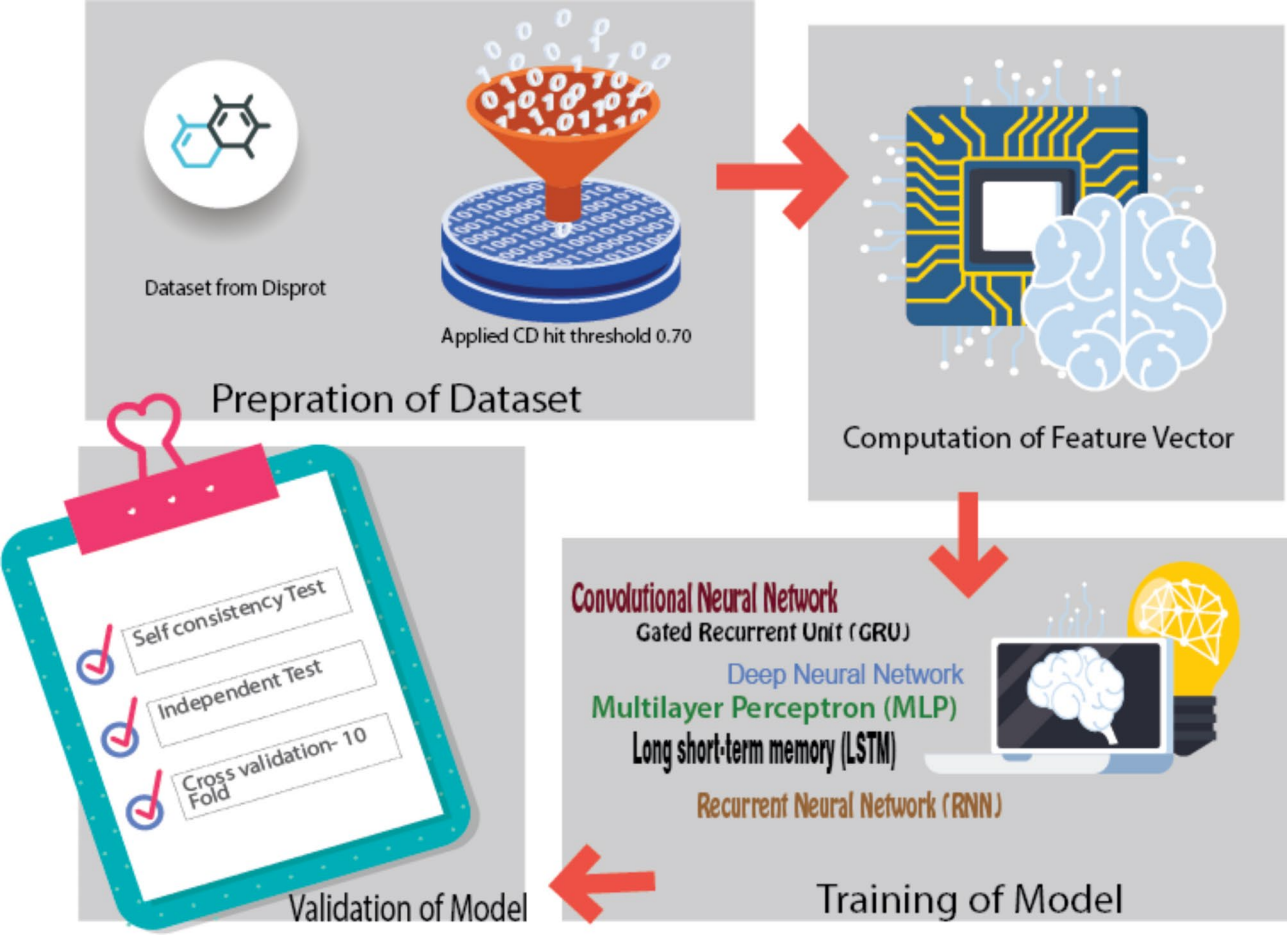
Architecture of Proposed Model.

A general workflow of our technique is provided in Fig. 1, which depicts the operation of an outline that was created expressly for the purpose. The next subsections will take a step-by-step, sequential approach to discussing each of the experimental steps that make up this suggested pipeline.

### Benchmark dataset

When trying to solve a bioinformatics problem, one of the most crucial measures to do is to collect a dataset of a good quality. In this work, we gathered all of the experimentally confirmed protein sequences from the Disprot database^4^, which is a massive database. DISPROT is one of the largest and most comprehensive databases of intrinsically disordered proteins (IDPs). It offers a vast amount of data that can be useful for generating hypotheses and identifying potential IDP candidates. DISPROT has been manually curated from the literature. The number of distinct articles used to extract the incorporated annotations is 3468 in the 2023_06 release of DISPROT. On the basis of the degrees to which they participated in transcription activation and articulation, the extracted sequences were divided into two distinct classes: ordered proteins and disordered proteins. We also extracted disordered sequences by taking into account intron, exon, and centromeric sequences but eliminating positive samples from the consideration. Following that, the CD-HIT^30^ have utilized in order to disbar out the paired samples whose similitude were determined to be greater than 70%.

The original dataset contains a total of 1446 different protein samples. There are 723 ordered protein samples and 723 disordered protein samples for the purpose of identifying disordered lipid binding residues. A 10-fold cross validation was carried out, for which the entire dataset was first randomly segmented into 10 subsets. The training procedure was carried out with a constant ratio of nine to one between the training set and the validation set, with the sets being rotated periodically.

## Computation of feature vector

We are dealing with a sequence of telomeres in this research paper, and we are utilizing two widely used techniques: composition-specific and position-variant, for the extraction of features from the sequences. Some of the components of the methods are described here.

## Position relative incidence matrix

The significant method to expose the hidden characteristics of the protein is dispersion of amino acid residues in the polypeptide chain. To record all the residue’s positions and their correlation we use matrix that turn into a complex pattern that exists due to residue placement. Each polypeptide chain considers twenty different amino acid residues. This matrix sets up 20 by 20 grid and referred as positional residue interaction matrix, which is usually use as PRIM. The main purpose of this grid is to estimate positional information for proteins.

$$R_{{PRIM}} = \left[ {\begin{array}{*{20}c} {R_{{1 \to 1}} } & {R_{{1 \to 2}} } & \cdots & {R_{{1 \to y}} } & \cdots & {R_{{1 \to 20}} } \\ {R_{{2 \to 1~}} } & {R_{{2 \to 2}} } & \cdots & {R_{{2 \to y}} } & \cdots & {R_{{2 \to 20}} } \\ \vdots & \vdots & {} & \vdots & {} & \vdots \\ {R_{{x \to 1}} } & {R_{{x \to 2}} } & \cdots & {R_{{x \to y}} } & \cdots & {R_{{i \to 20}} } \\ \vdots & \vdots & {} & \vdots & {} & \vdots \\ {R_{{A \to 1}} } & {R_{{A \to 2}} } & \cdots & {R_{{A \to y}} } & \cdots & {R_{{A \to 20}} } \\ \end{array} } \right]$$

On the basis of the relative position of jth residue the sum is calculated by ith residue which is represented by every g-element (Rij) in PRIM matrix. Hence, the final matrix consists of 400 coefficients. To reduce dimension complexity, the statistical moments are calculated to come up with a set, which is derived from the original 400 coefficient matrix, consists of 30 listed features.

## Reverse position relative incidence matrix (RPRIM)

Just as previous method, the Reverse Position Relative Incidence Matrix (RPRIM) goes one step further in revealing hidden information in sequences that have homologous idiosyncratic features. The input sequence on which similarity was calculated is reversed for RPRIM calculation. The following is the matrix generated from this process.

$$R_{{RPRIM}} = \left[ {\begin{array}{*{20}c} {Q_{{1 \to 1}} } & {Q_{{1 \to 2}} } & \cdots & {Q_{{1 \to y}} } & \cdots & {Q_{{1 \to 20}} } \\ {Q_{{2 \to 1~}} } & {Q_{{2 \to 2}} } & \cdots & {Q_{{2 \to y}} } & \cdots & {Q_{{2 \to 20}} } \\ \vdots & \vdots & {} & \vdots & {} & \vdots \\ {Q_{{x \to 1}} } & {Q_{{x \to 2}} } & \cdots & {Q_{{x \to y}} } & \cdots & {Q_{{i \to 20}} } \\ \vdots & \vdots & {} & \vdots & {} & \vdots \\ {Q_{{A \to 1}} } & {Q_{{A \to 2}} } & \cdots & {Q_{{A \to y}} } & \cdots & {Q_{{A \to 20}} } \\ \end{array} } \right]$$

Thirty unique features were taken and then statistical moments applied over all features, ten features for every moment; raw, central, and Hahn. As the dimensionality PRI at 400 coefficients the RPRIM remains same.

## Frequency vector (FV)

In the specific sequence the major information about the dispersal of residues is provided by frequency vector. In that specific sequence it calculates the frequency of every amino acid. The distributional and compositional information about the sequences is ensure through frequency vector.

3 $$FV = [f_{1} ,f_{2} ,f_{3} , \ldots \:,f_{{20}} ]$$

With twenty coefficient FV is a vector which is used to compute the frequency of each amino acid residue in a sequence.

### Accumulative absoute position incidence vector

In other words, the FV characterizes the hidden properties of a sequence composition and encapsulates the distributional data for every amino acid residue in the sequence. But naturally, this data will not disclose the amino acid residue’s relative positions in FV. Therefore, we get four quarters by dividing relative positional information through AAPIV. On the basis of twenty native amino acids the calculation is made, as follows.

4 $$K = [\forall _{1} ,\forall _{{2,}} \forall _{{3,}} , \ldots ,\forall _{n} ]$$

Where AAPIV’s *i*^*th*^ section is calculated as

5 $$\:{\forall\:}_{i}={{\varSigma\:}^{n}}_{k=1}\:{\beta\:}_{k}$$

Where k is some position which was uniformly selected at random concerning given nucleotide. The explicit term for the AAPIV, I, sums over all of the locations in which the ith nucleotide occurs.

## Reverse accumulative absolute position incidence vector - RAAPIV

AAPIV and RAAPIV are similar but the main difference, which is the fact, is by using the reverse sequence of original sample the output vector is generated by RAAPIV. With this reversal, extracting much information on positional information is possible. As such, one can be able to reveal features, which are hidden, in the sequences deeply. The vector’s representation is as follows.

6 $$RAAPIV = [n_{1} ,n_{{2,}} n_{{3,}} , \ldots ,n_{m} ]$$

## Statistical moments

The genomic data is converted into fixed-size vectors by using the statistical moments. Every moment provides unique information about the nature of data. Researchers have probed the moments for a variety of distributions to see if they are fit for this procedure. The feature set will include raw, central, and Hahn moments of the genomic data, thus establishing essential input vector’s elements for model. It was noted that the genomic and proteomic sequences share base positions that their features depend upon. As a result, there have been developed several computational and mathematical models for the study of nucleotide base’s correlated positioning in genomic sequences for feature vector. It’s essential to proceed with a reliable and consistent set of feature^24^. Genomic sequences are converted into a two-dimensional matrix S’ of size k*k having similar information to S but in a two-dimensional vector form since two-dimensional data is required by Hahn moments^25^.

7 $${\text{k}} = \sqrt n$$

8 $$S^{\prime } = \left| {S_{{11}} S_{{12}} \ldots S_{{1n}} S_{{21 \ldots }} S_{{22 \ldots }} \ldots S_{{2n \ldots }} S_{{n1}} S_{{n2}} \ldots S_{{nn}}} \right|$$

Fixed-size feature vector is created from the square matrix obtained and statistical moments, which helps in reducing its dimensionality^26^.

For this study, raw moments, Hahn moments, and central moments are employed. Below the expression indicates computation of raw moments of order a + b:.

9 $$\:{U}_{ab}={{\varSigma\:}^{n}}_{e=1}{{\varSigma\:}^{n}}_{f=1}\:{e}^{a}{f}^{b}\delta\:ef$$

Up to order 3 of Moments, significant information is embedded in the following sequences, which are U00, U10, U11, U20, U02, U21, U12, U03 and U30. Calculating the central moments also requires that the centroid (x, y) is calculated^23^. The central point of the data is centroid. This will be used to calculate the central moments:

10 $$v_{{ab}} = \Sigma ^{n} _{{e = 1}} \Sigma ^{n} _{{f = 1)~}} \left( {e - \underset{\raise0.3em\hbox{$\smash{\scriptscriptstyle-}$}}{x} } \right)^{a} \left( {f - \underset{\raise0.3em\hbox{$\smash{\scriptscriptstyle-}$}}{y} } \right)^{b} \delta ef$$

The Hahn moments were computed using a discretized input square grid; it offers data regularity and reversibility, since using inverse Hahn moments the original data can be reconstructed.

Because the Hahn moments are reversible, any information, which is transformed from original sequences, keeps and utilized to reconstruct the feature vector of model. Following is the Hahn moment’s calculation.

11 $$\:{h}_{n}^{x,y}\left(p,Q\right)=(Q+{V-1)}_{n}(Q{-1)}_{n}\times\:{{\varSigma\:}^{n}}_{z=0}{\left(-1\right)}^{z}\frac{\left({-n)}_{z}\right({-p)}_{z}({2Q+x+y-n-1)}_{z}}{\left({Q+y-1)}_{z}\right(Q{-1)}_{z}}\frac{1}{z!}$$

The Pochhammer notation is defined by the following Eq. (11) and Gamma operator, which is well described by Akmal et al.^27^. Applying above equation, usually with the help of coefficient, defined as follows, the coefficient for Hahn moments are normalized.

12 $$\:{H}_{pq}=\:{{\varSigma\:}^{G-1}}_{j=0}{{\varSigma\:}^{G-1}}_{i=0}\:{\delta\:}_{pq}{{h}^{a,b}}_{p}\left(j,Q\right)\:{{h}^{a,b}}_{q}\:\left(i,Q\right),\:\:\:\:\:\:m,n=\text{0,1},2,\:\dots\:,\:Q-1$$

### Convolutional neural network

**Fig. 2:**
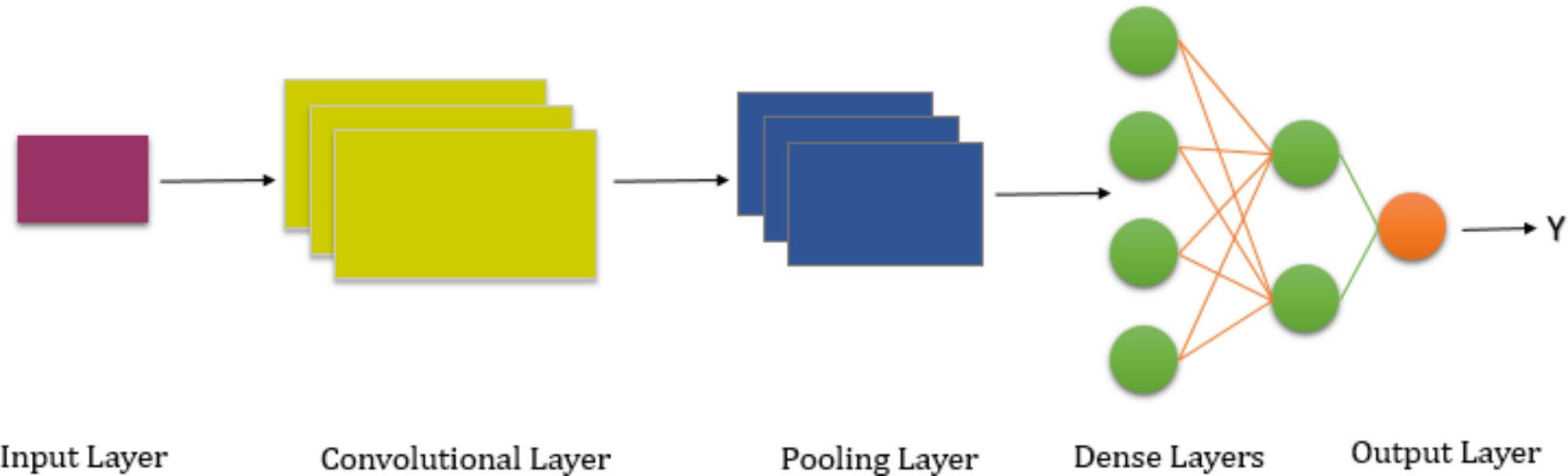
Workflow of Convolutional Neural Network.

A Convolutional Neural Network (CNN) is a deep learning technique that is used to process and analyze data that has a grid-like structure, such as photos, videos, and time series data. CNNs are very effective in computer vision approaches such as image categorization, entity classification, and picture sectionalization. CNNs distinguish themselves by their capacity to automatically learn and derive hierarchical representations from incoming data. Convolutional layers, which execute convolutions between input data and a collection of learnable filters or kernels, are used to do this. Local patterns and characteristics, such as edges or textures, are captured by these filters at various spatial positions within the input. CNNs may train to recognize many features at the same time by employing multiple filters. Pooling layers, which down-sample the feature maps created by the convolutional layers, are also included in CNNs. Pooling aids in reducing the spatial dimensions of the data while retaining the most important properties. This results in a more compact representation, allowing the network to concentrate on the most significant features of the material as shown in Fig. 2. The retrieved characteristics are then sent into fully connected layers, which are standard neural network layers^31–33^.

### Deep neural network (DNN)

**Fig. 3:**
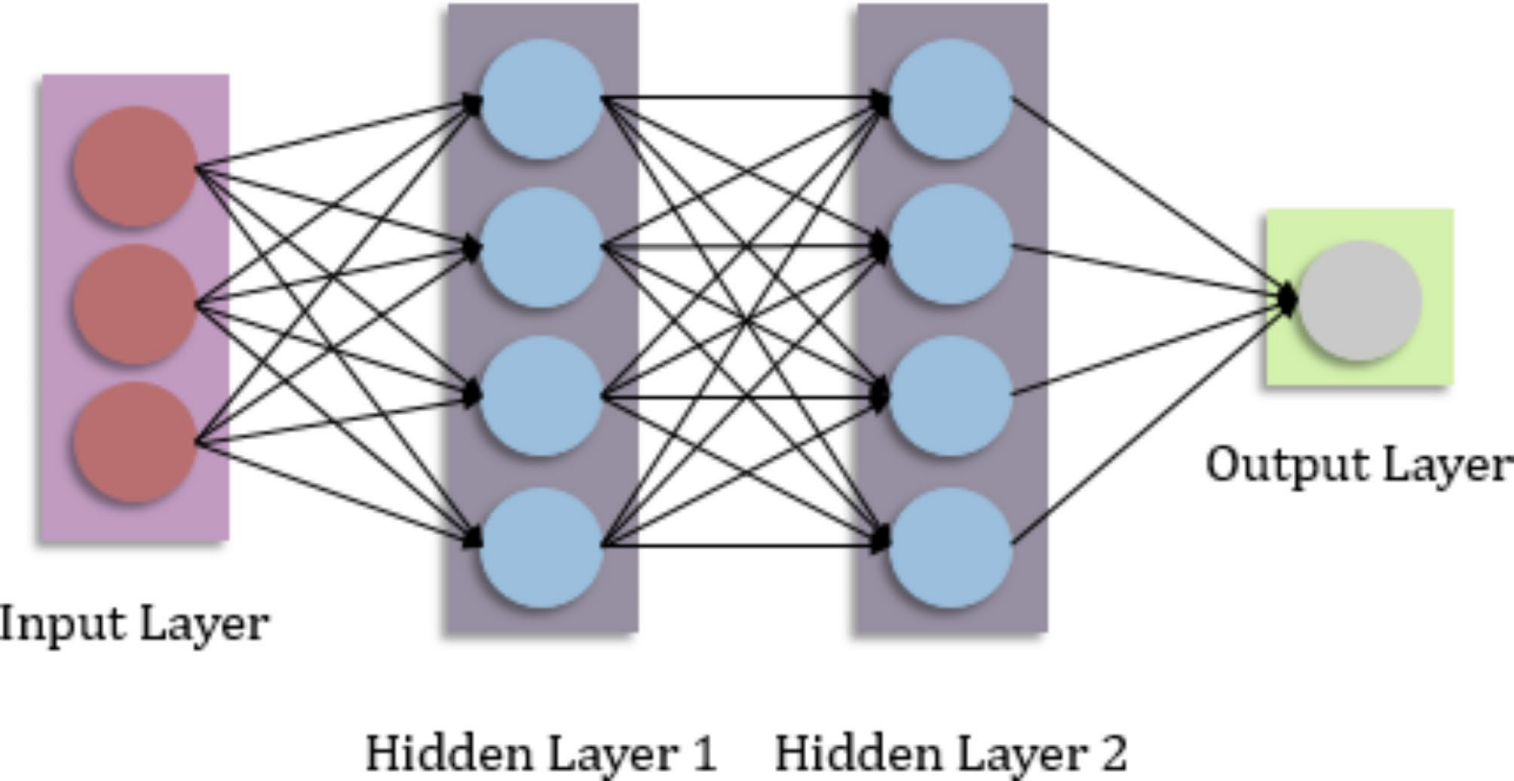
Structure of Deep Neural Network.

A Deep Neural Network (DNN) is a form of artificial neural network made up of numerous layers of linked nodes called neurons^34^. DNNs are built to learn and express complicated relationships in data using several layers of non-linear transformations. The raw input data is received by the input layer of a DNN and then passed via a succession of hidden layers. Each buried layer is made up of several neurons that process the incoming input. The output of one layer is used as the input for the next layer, enabling for the extraction of higher-level features and representations to be done in stages^35^. The capacity of DNNs to learn abstract and hierarchical representations of incoming data is their strength. As input flows through the network, each layer learns to extract increasingly complex and significant information from the output of the preceding layer. DNNs may capture complicated patterns and relationships in data using this hierarchical representation. Backpropagation is a technique used to train a DNN in which the network changes its weights and biases to minimize the discrepancy between the expected and actual output^36^. Typically, this optimization process is carried out using gradient descent methods, which update the network parameters based on the gradients of the loss function with respect to the parameters, illustrated in Fig. 3.

### Multilayer perceptron (MLP)

**Fig. 4:**
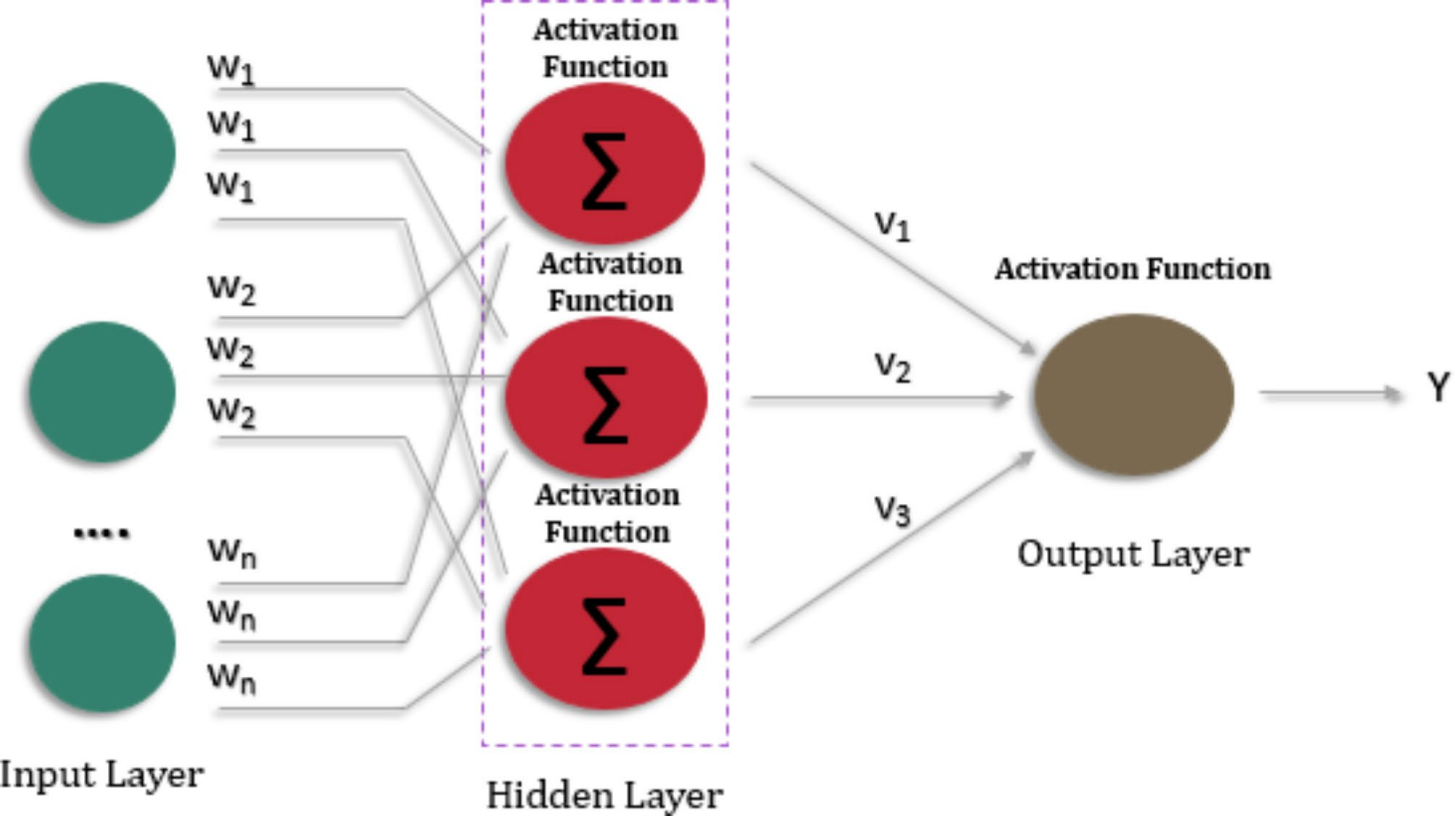
Representation of Multi-Layer Perceptron.

A Multi-Layer Perceptron (MLP) is a feedforward artificial neural network with numerous layers of linked neurons. It is one of the most basic and extensively used neural network topologies. MLPs are mostly utilized for supervised learning applications like classification and regression. The network in an MLP is made up of an input layer, one or more hidden layers, and an output layer. Each layer is made up of several artificial neurons, also known as perceptron, shown in Fig. 4. The neurons in one layer are completely linked to the neurons in the layers above and below. The weights associated with neural connections determine the strength of the connection. During an MLP’s forward pass, input data is sent into the input layer, and calculations are carried out layer by layer. Each neuron in a hidden layer gets input from neurons in the preceding layer, applies an activation function to the weighted sum of its inputs, and generates an output. This procedure is continued until the output layer is reached, which results in the network’s final output. MLPs are distinguished by non-linear activation functions such as sigmoid, tanh, or ReLU (Rectified Linear Unit), which add non-linearity into the network and allow it to learn complicated data correlations.

### Recurrent neural network (RNN)

**Fig. 5:**
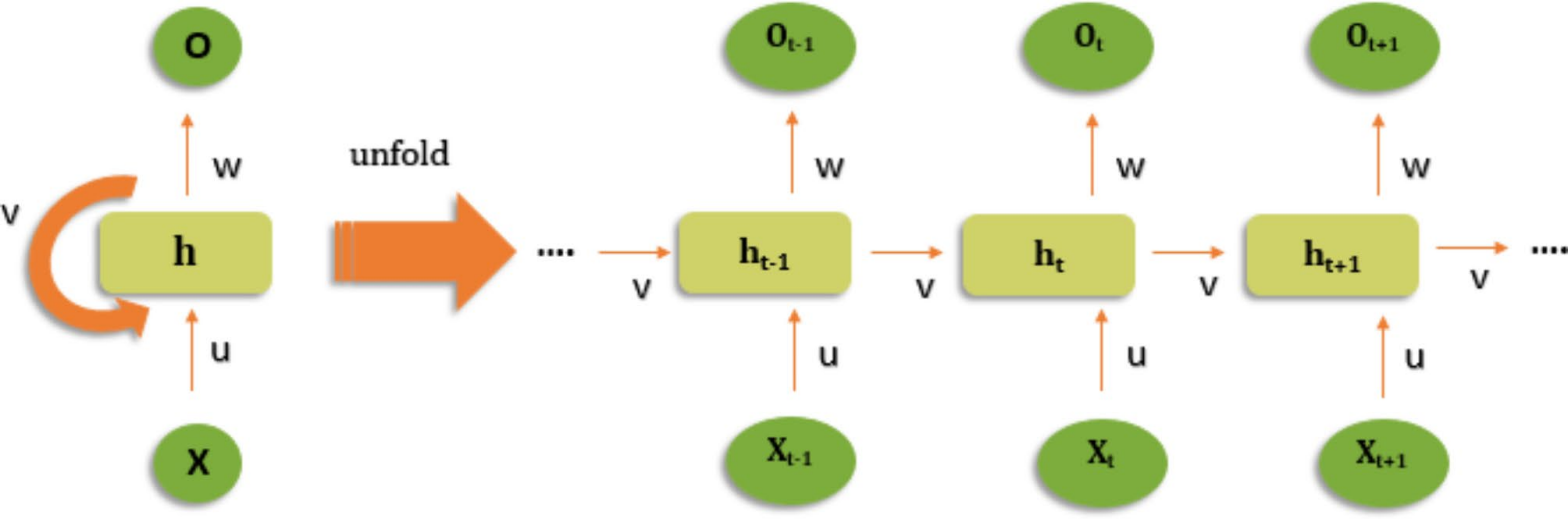
Working Diagram of Recurrent Neural Network.

A Recurrent Neural Network (RNN) is a form of artificial neural network that uses recurrent connections between neurons to process sequential input, illustration Fig. 5. RNNs, as opposed to feedforward neural networks, feature feedback connections that allow them to save and use information from earlier time steps or inputs^37^. RNNs are distinguished by their capacity to grasp temporal relationships and handle sequences of varied lengths. As a result, they are well-suited to jobs requiring time series data, natural language processing, speech recognition, and other sequential data analysis. Each neuron in an RNN has an internal memory state that acts as a hidden state or context vector. The network takes an input and mixes it with the previous hidden state to create a new hidden state at each time step^38^. Because of this recursive feedback loop, information may remain and flow through the network, allowing it to represent and capture long-term relationships in sequential data. Depending on the job, the output of an RNN can be generated at each time step or at the end. In sequence classification, for example, the output is often generated at the final time step, summarizing the whole sequence. In sequence creation, such as text generation, the network can create outputs at each time step, progressively generating a sequence.

### Long short-term memory (LSTM)

**Fig. 6:**
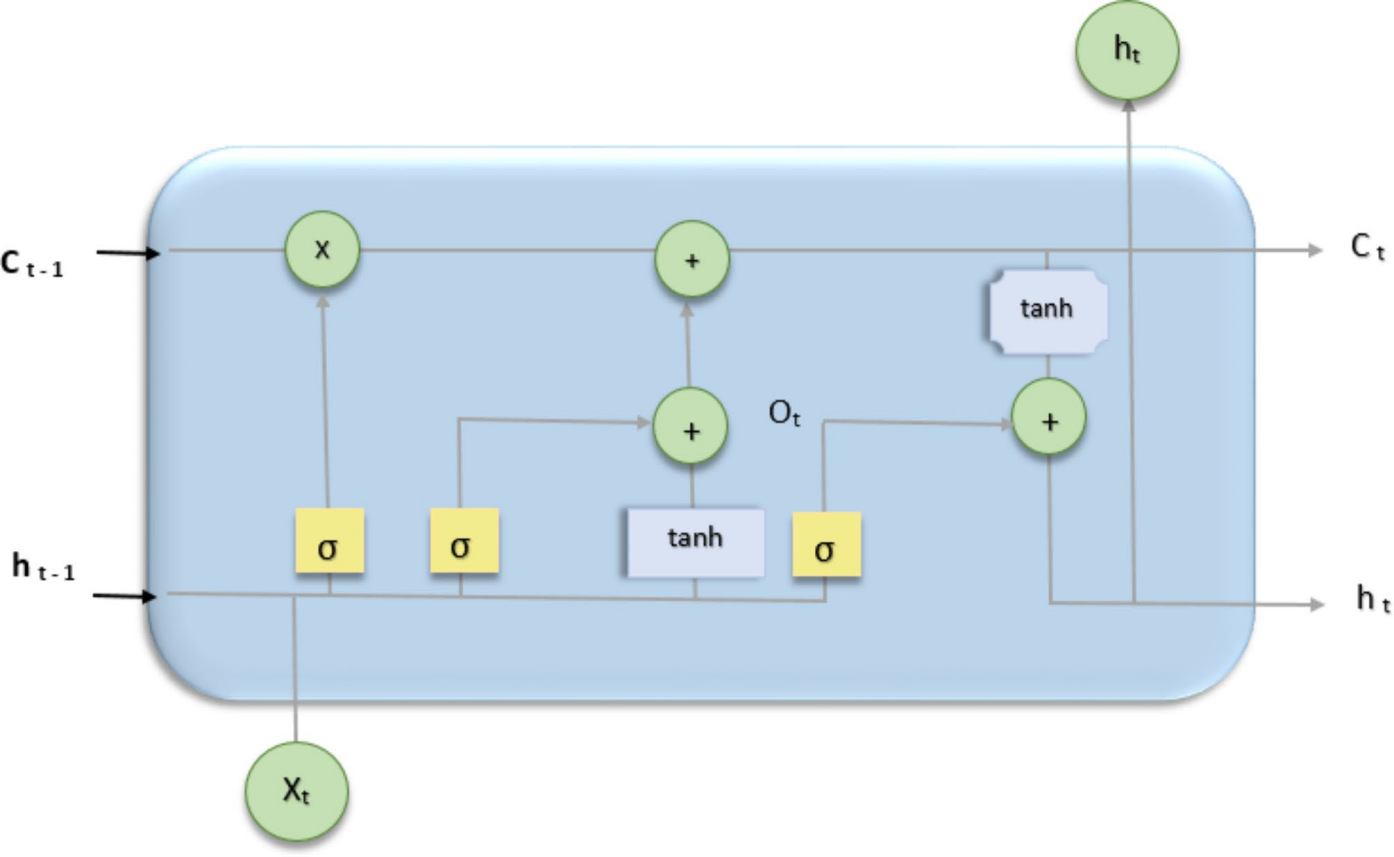
Model of Long short-term memory.

Long Short-Term Memory (LSTM) is a Recurrent Neural Network (RNN) version developed to solve the difficulties of collecting long-term relationships in sequential input represented in Fig. 6. To address the vanishing gradient problem and allow RNNs to learn and store knowledge over longer time intervals, LSTMs were invented. The memory cell is the basic building element of an LSTM, and it is made up of multiple components: an input gate, a forget gate, a cell state, and an output gate. These components collaborate to control the flow of information within the LSTM. The input gate regulates how much of the incoming data should be kept in the cell level at each time step. It applies a sigmoid activation function to the current input and the prior concealed state, yielding a number between 0 and 1 that indicates the current input’s contribution to the cell state. The forget gate determines which cell state information should be deleted. It analyses the current input and the prior concealed state, using a sigmoid activation function to determine how much of the cell state should be remembered and how much should be lost. The cell state acts as the LSTM’s long-term memory. It is updated by combining the prior cell state with the input gate’s contribution and deleting the useless information indicated by the forget gate. Finally, at each time step, the output gate decides the output of the LSTM. It takes into account the updated cell state and the current input before producing a value between 0 and 1. This value is multiplied by the cell state after a tanh activation function is applied, yielding the LSTM output for the current time step.

### Gated recurrent unit (GRU)

**Fig. 7:**
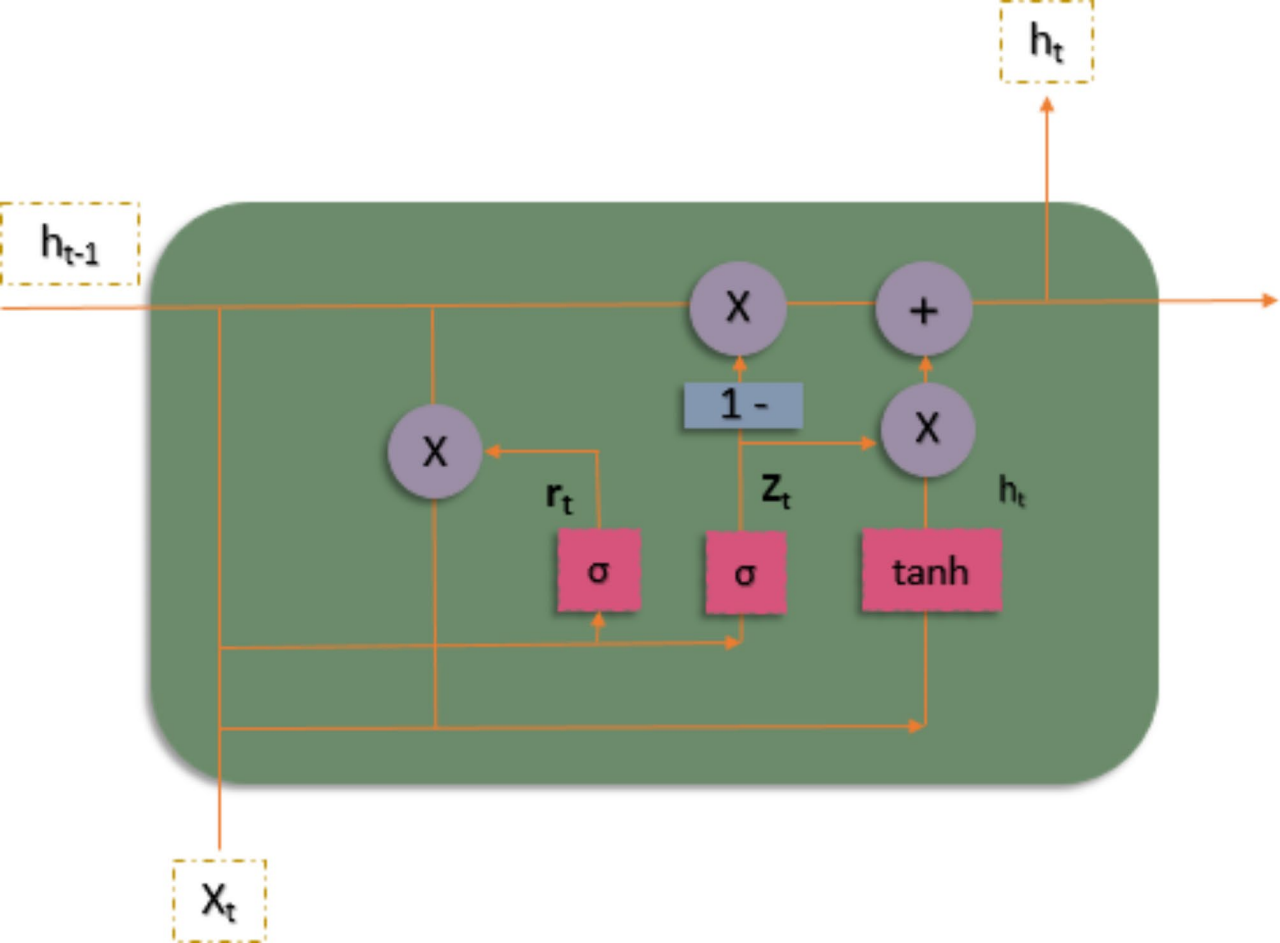
Architecture of Gated Recurrent Unit.

GRU is an abbreviation for “Gated Recurrent Unit.” It is a recurrent neural network (RNN) architecture that was developed to improve on existing RNNs. GRUs are intended to overcome the vanishing gradient problem that can arise while training deep neural networks, particularly ones with lengthy sequences. The use of gating mechanisms allows the network to selectively update and reset its hidden state, which is a critical property of GRUs. The gating mechanism assists the network in retaining useful information from prior time steps while discarding irrelevant data. This enables GRUs to more efficiently capture long-term relationships in sequential data. A GRU unit typically has two major gates: an update gate and a reset gate as shown in Fig. 7. The update gate controls how much of the old hidden state is kept and how much of the new candidate hidden state is introduced. The reset gate regulates how much of the foregoing data should be erased. GRUs can efficiently collect important information and propagate it over time by adaptively updating and resetting the hidden state. GRUs have a simpler design with fewer parameters than other forms of RNNs, such as the basic RNN or the more complicated LSTM.

### The proposed approach

In this study, the proposed system consists of combination of advanced feature formulation techniques such as relative positioning with a Convolutional Neural Network (CNN) to analyze sequences. The feature formulation involves two widely used techniques: composition-specific and position-variant, which extract detailed features from the sequences, capturing both the compositional and positional information of amino acid residues. The core of our feature extraction process includes the Position Relative Incidence Matrix (PRIM) and the Reverse Position Relative Incidence Matrix (RPRIM). PRIM captures the positional interaction among amino acid residues in a polypeptide chain, resulting in a 20 × 20 matrix that provides a comprehensive positional overview. The matrix records the positions and correlations of residues, generating 400 coefficients that are then reduced to a manageable set of 30 features through statistical moments. RPRIM extends this concept by reversing the sequence, revealing hidden homologous features and ensuring no positional information is lost. This dual approach of PRIM and RPRIM ensures a thorough capture of both forward and reverse positional relationships. Additionally, we use the Frequency Vector (FV) to calculate the frequency of each amino acid residue in a sequence, providing essential compositional data. To further enhance the positional information, the Accumulative Absolute Position Incidence Vector (AAPIV) and its reverse (RAAPIV) are utilized. These vectors split the sequence into quarters and accumulate positional data, offering a refined view of residue distribution. The extracted features are then fed into a CNN, renowned for its proficiency in capturing spatial hierarchies in data. CNNs consist of multiple layers that convolve the input features, enabling the network to learn intricate patterns. The combination of CNN with our advanced feature formulation methods allows for the efficient processing of high-dimensional data, leading to superior predictive performance. Our CNN model, trained on these meticulously extracted features, has demonstrated remarkable accuracy and robustness. The integration of sophisticated feature extraction techniques with a CNN forms a powerful system for analyzing telomere sequences. By capturing both compositional and positional information, our system can identify subtle patterns that traditional methods might overlook.

### Performance assessment

After developing a machine learning computational model, it is critical to assess the model’s performance to determine how successfully it handled the given issue. This is accomplished through the use of several performance estimate methodologies, references, all of the preceding investigations make use of parameters. The usage of a parameter is determined by the sample class and the classification issue. The confusion matrix is used to generate performance evaluation metrics^39–42^. The right and wrong values for each class are stored in the confusion matrix. Comparisons are made between the confusion matrix results and the actual outcomes. Each column of the confusion matrix reflects the actual value for that class, whereas the rows of the matrix represent the anticipated class.

13 $$\:Accuracy=\:\frac{TP+TN}{TP+FP+FN+TN}$$

14 $$\:Sensitivity=\:\frac{TP}{TP+FP}$$

15 $$\:Specificity\:=\:\frac{TP}{FP+TN}$$

16 $$\:MCC=\:\frac{TP\:\times\:TN-FP\:\times\:FN}{\sqrt{\left[TP+FP\right]\left[TP+FN\right]\left[TN+FP\right]\left[TN+FN\right]}}$$

## Results and discussion

### Comparison of different deep learning models via 10-fold CV

**Fig. 8:**
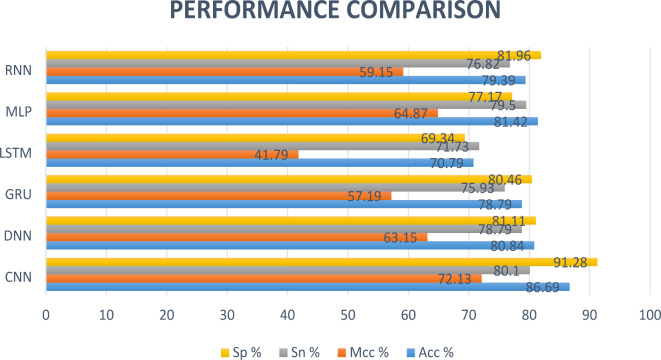
Illustration of different deep learning models for comparison.

**Fig. 9:**
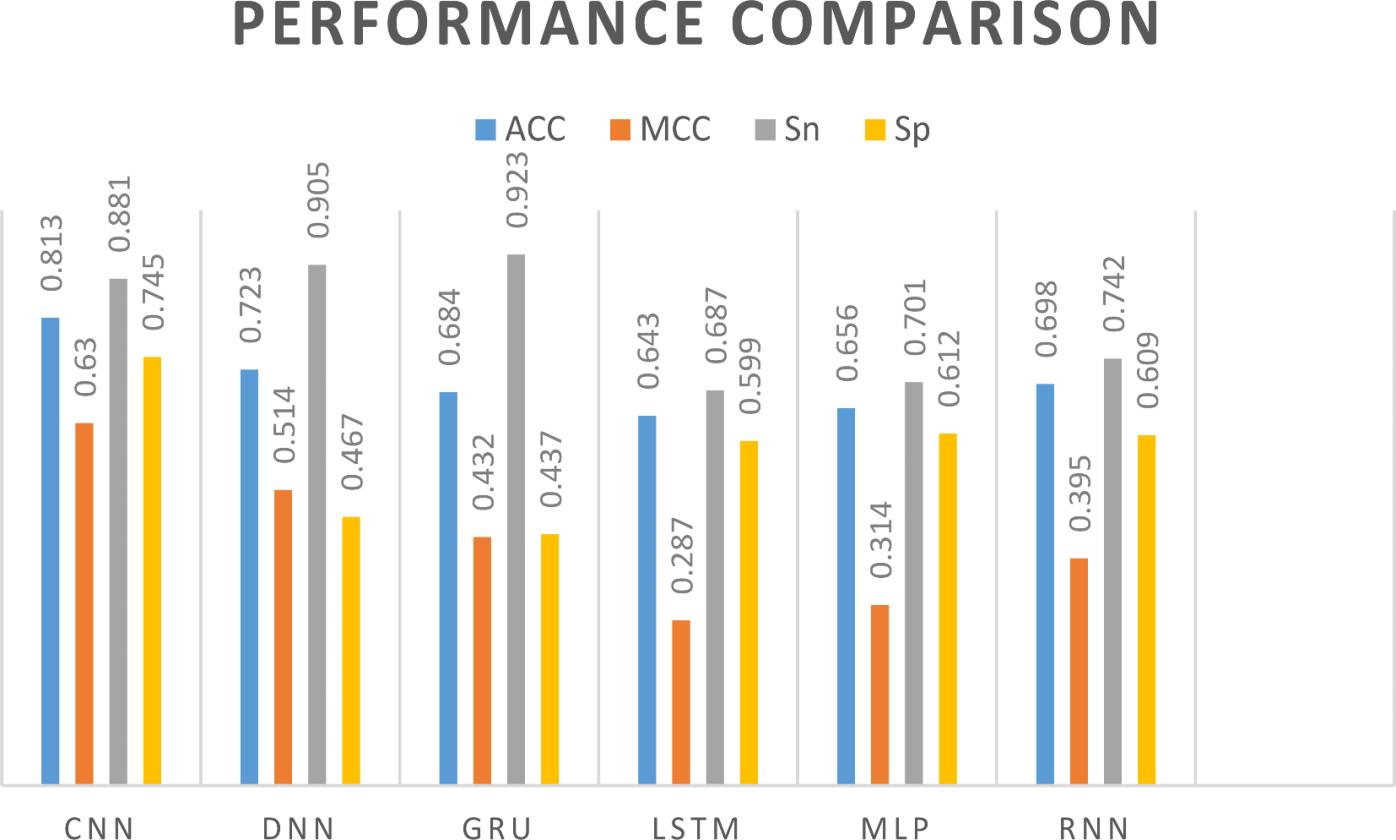
ROC curves of different deep learning models on benchmark (A) and IND (B) datasets.

We feed the acquired features into various neural network models for prediction, including CNN, DNN, GRU, LSTM, and RNN achieved ACC of 0.866, 0.808, 0.787, 0.707, 0.814 and 0.793, illustrated in Fig. 8, respectively. In Fig. 9 (A), the ROC curves of the dataset are displayed in accordance with the various prediction models. Table 1 contains a description of the results of the predictions. When compared to the best conventional classifiers, we can demonstrate that deep learning models have higher learning skills and better prediction outcomes than general classifiers. After Comparison we see that, the performance evaluation of our model’s predictions is more accurate. In Table 1, bold highlighted values are the highest ones from all used models.

**Table 1:** Performance comparison of deep learning models on dataset by tenfold cross-validation.

Method	ACC	MCC	Sn	Sp	AucRoc	F1
CNN	***0.866***	***0.721***	***0.801***	***0.912***	***0.925***	***0.831***
DNN	0.808	0.631	0.787	0.811	0.887	0.787
GRU	0.787	0.571	0.759	0.804	0.851	0.752
LSTM	0.707	0.417	0.717	0.693	0.790	0.687
MLP	0.814	0.648	0.795	0.771	0.892	0.774
RNN	0.793	0.591	0.768	0.819	0.874	0.763

### Comparison of different deep learning models via independent test

**Fig. 10:**
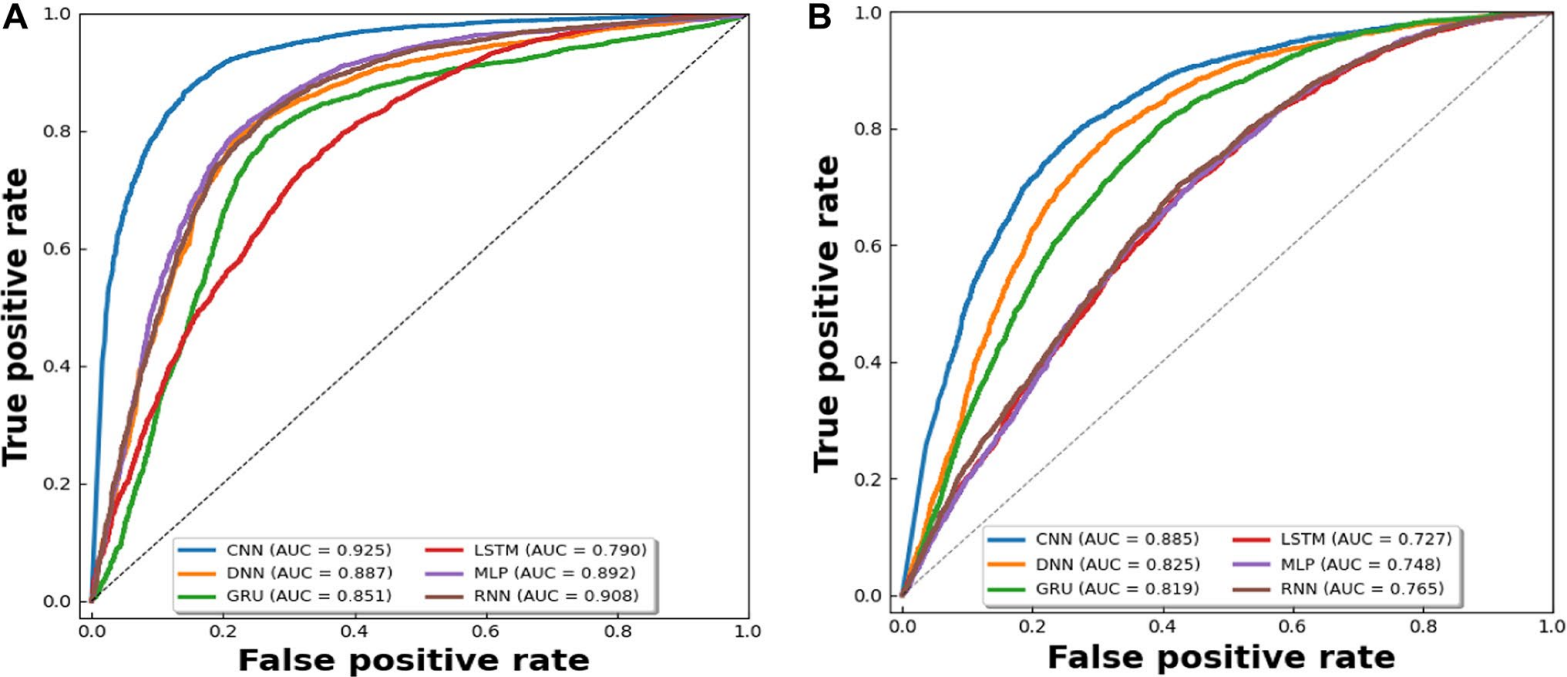
ROC curves of different deep learning models on benchmark (A) and IND (B) datasets.

We feed the acquired features into various neural network models for independent testing, including CNN, DNN, GRU, LSTM, and RNN achieved ACC of 0.813, 0.723, 0.684, 0.643, 0.656 and 0.698, illustrated in Fig. 10, respectively. In Fig. 9 (B), the ROC curves of the dataset are displayed in accordance with the various prediction models. Table 2 contains a description of the results of the predictions. After Comparison we see that, the performance evaluation of our model’s predictions is more accurate. In Table 2, bold highlighted values are the highest ones from all used models.

**Table 2:** Performance comparison of deep learning models on dataset by independent testing.

Method	ACC	MCC	Sn	Sp	AucRoc	F1
CNN	***0.813***	***0.63***	***0.908***	***0.745***	***0.885***	***0.861***
DNN	0.723	0.514	0.905	0.467	0.825	0.757
GRU	0.684	0.432	0.889	0.437	0.819	0.681
LSTM	0.643	0.287	0.687	0.599	0.727	0.615
MLP	0.656	0.314	0.701	0.612	0.748	0.621
RNN	0.698	0.395	0.742	0.609	0.765	0.675

### Comparison of different deep learning models via self-consistency test

**Fig. 11:**
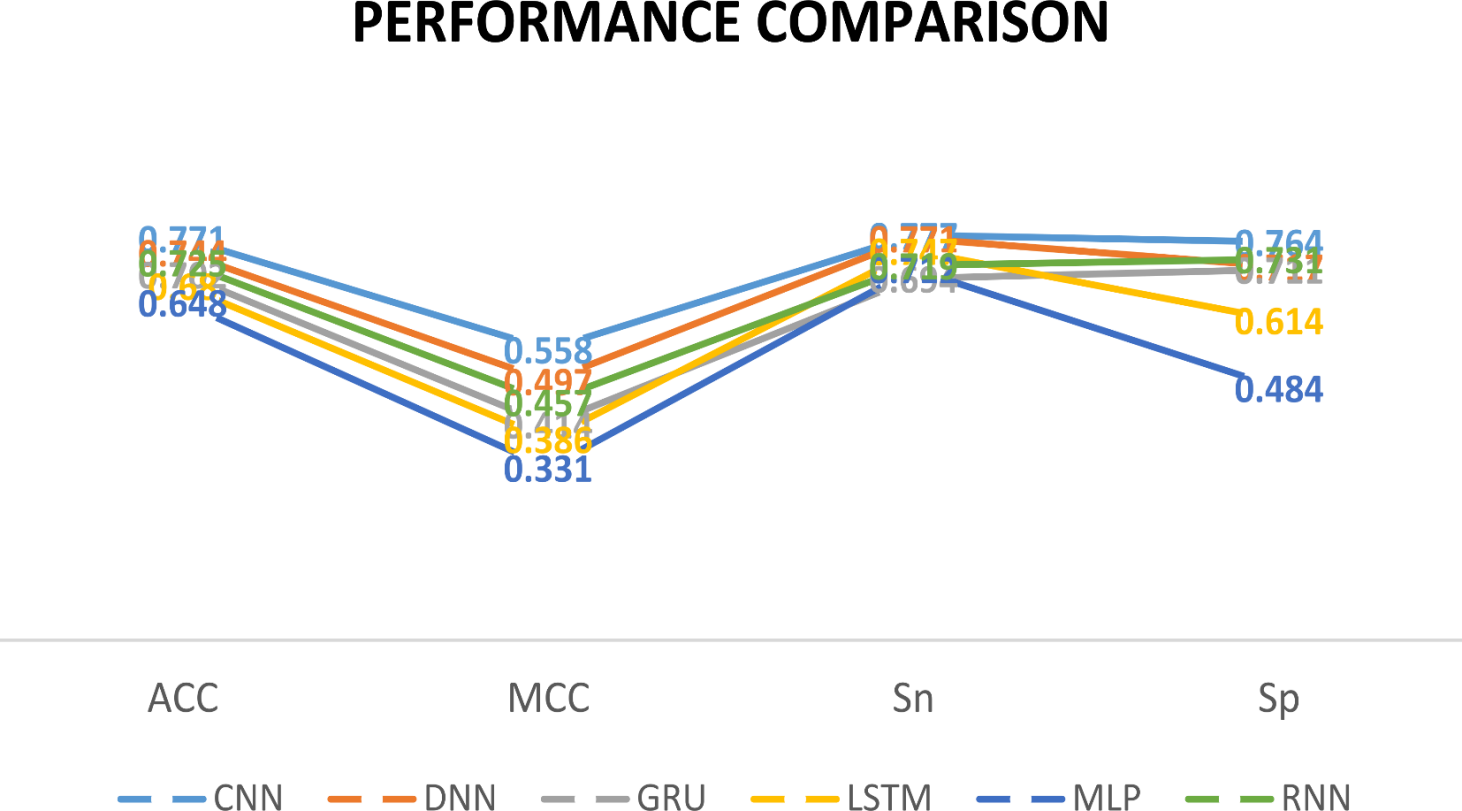
Illustration of different deep learning models for comparison on self-consistency dataset.

**Fig. 12:**
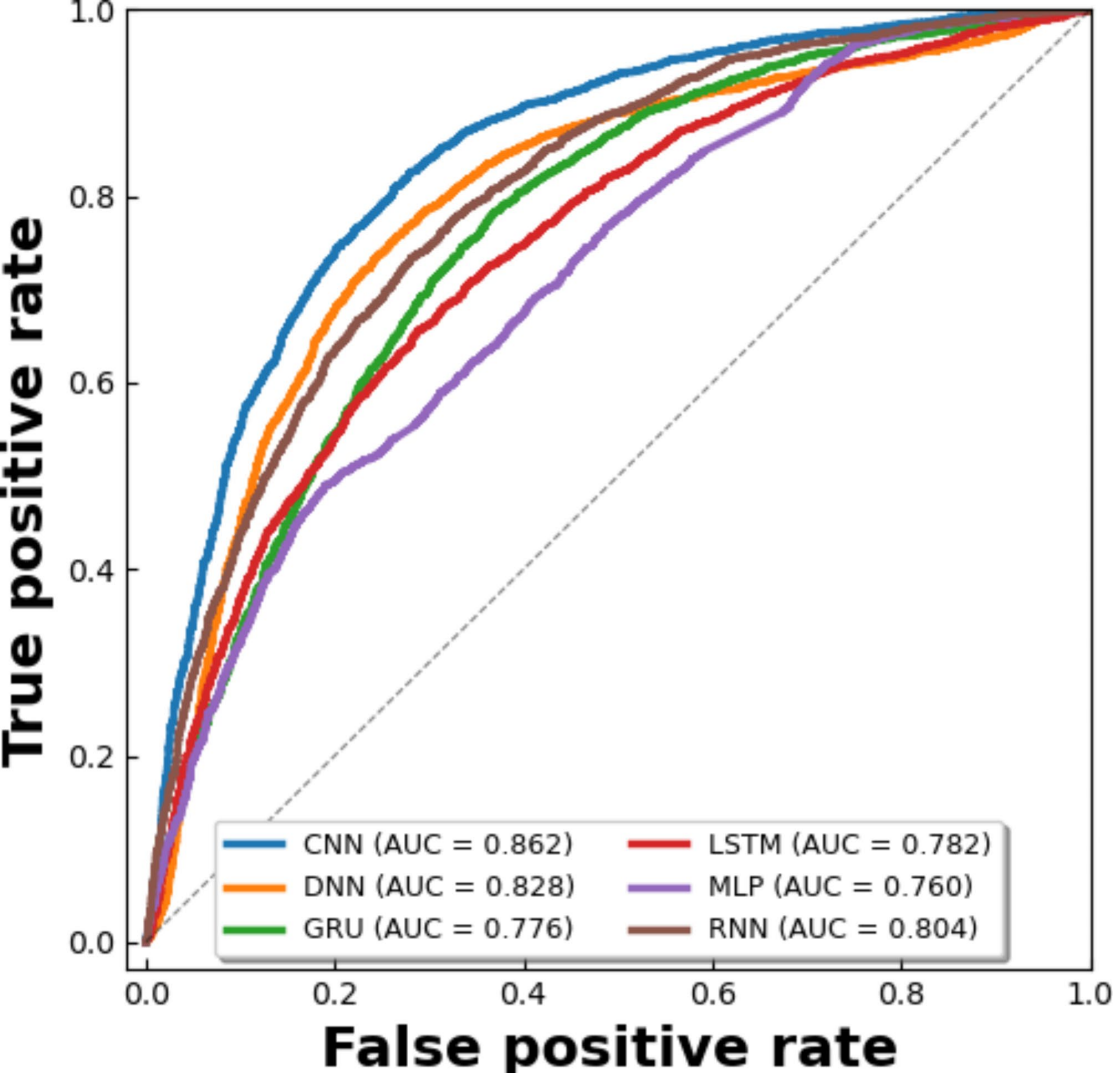
ROC curves of different deep learning models with self-consistency test.

We feed the acquired features into various neural network models for self-consistency testing, including CNN, DNN, GRU, LSTM, and RNN achieved ACC of 0.771, 0.744, 0.702, 0.680, 0.628 and 0.725, illustrated in Fig. 11, respectively. In Fig. 12, the ROC curves of the dataset are displayed in accordance with the various prediction models. Table 3 contains a description of the results of the predictions. After Comparison we see that, the performance evaluation of our model’s predictions is more accurate.

**Table 3:** Performance comparison of deep learning models on dataset by self-consistency testing.

Method	ACC	MCC	Sn	Sp	AucRoc	F1
CNN	***0.771***	***0.558***	***0.777***	***0.764***	***0.862***	***0.765***
DNN	0.744	0.497	0.771	0.717	0.828	0.732
GRU	0.702	0.414	0.694	0.711	0.776	0.689
LSTM	0.680	0.386	0.747	0.614	0.782	0.642
MLP	0.648	0.331	0.712	0.484	0.760	0.618
RNN	0.725	0.457	0.719	0.731	0.804	0.718

### Boundary visualization

**Fig. 13:**
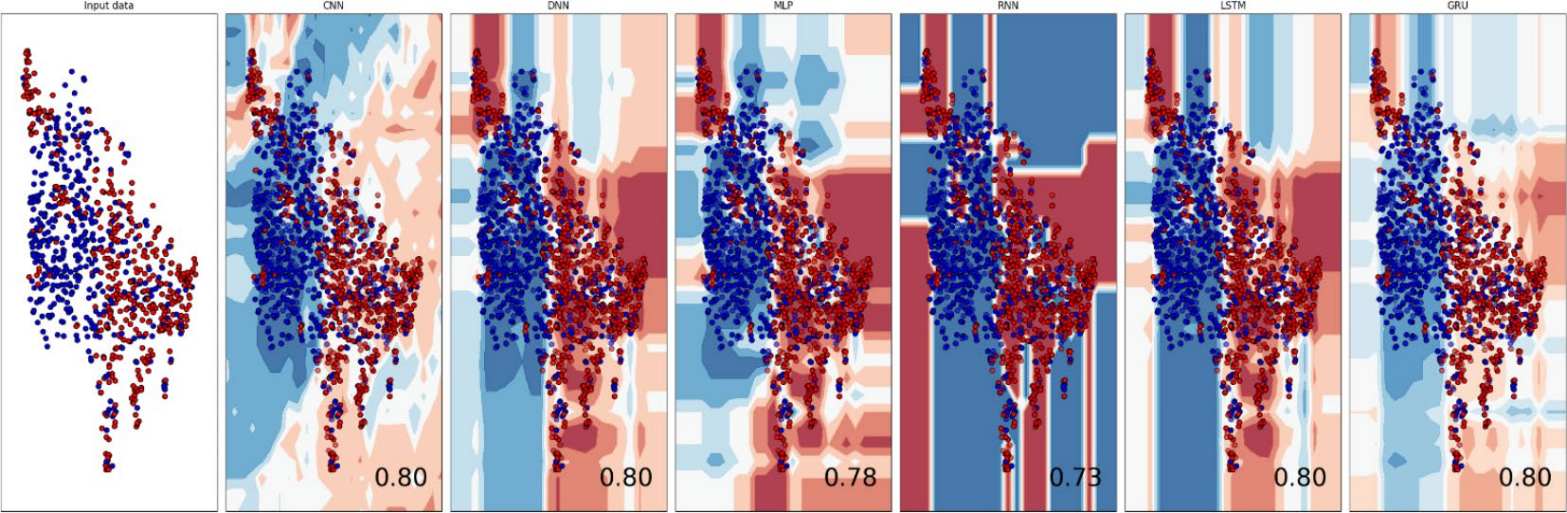
Boundary visualization of ensemble models: (**A**) Input data. **(B**) CNN. (**C**) DNN. (**D**) MLP. (**E**) RNN. (**F**) LSTM. (**G**) GRU.

A boundary visualization graph, also known as a decision boundary plot, is a graphical representation that shows how a binary classification model divides a dataset into multiple groups^43,44^. The purpose of binary classification is to categorize data items into one of two classes or categories depending on their attributes as shown in Fig. 13. The decision boundary is the dividing line or surface in the feature space that divides these classes (ordered and disordered regions). The boundary visualization graph reveals important information about how well the model performs and how it differentiates the classes. It aids in identifying areas where the classes overlap or are difficult to differentiate, as well as areas where the model’s predictions may be unclear. Analysts and data scientists can acquire a better grasp of the model’s behavior and evaluate its performance across the feature space by visualizing the decision boundary.

A classification model is trained to partition a sample space into distinct classes. Such that whenever a sample is mapped onto the space the corresponding class could be identified. Boundary visualizations aims to visualize the partitioning of these subspaces. The red hue signifies positive sample space while blue represents negative samples. The visualization can assist biologists to infer positive or negative samples. In case numerical representation of an uncategorized sequences is mapped into red subspace then it will be confidently inferred as a positive sample by the biologist.

## Conclusion

The research discusses intrinsically disordered proteins (IDPs), which lack a defined three-dimensional structure but exhibit dynamic and structural organization. The scope of the study is limited to computational classification of intrinsically disordered proteins. It aims to serve as a tool for biologists that can be used to quickly identify disordered proteins given a primary sequence without the use of time and cost intensive in-vivo or in-vitro analyses. Cross-validation test is a rigorous test in which the data is partitioned into number of folds. In each iteration one disjoint partition is left out while the model is trained on the rest of partitions. After training the model is tested with the left-out partition which is unknown data for the trained model. This is repeated for all the disjoint partitions. So, virtually the model is being rigorously tested to identify unknown data spanning over the whole data set. It is left to the biologist to discover uncategorized sequences and use the model to identify whether the protein corresponding to the primary sequence is disordered or not. However, it does not fall within the scope of our work to analytically collect new sequences and use them to verify our model. The flexibility of IDPs suggests that entropy-driven motions are crucial for their function. Interactions between IDPs and specific binding partners lead to disorder-to-order transitions, vital for their function. Biophysical characterization of IDPs involves experimental and theoretical methods. A new deep learning-based method is introduced to distinguish between disordered and ordered regions within IDPs, achieving high accuracy in identifying both types of regions. This approach represents a novel application of bioinformatics to IDPs.

## Data Availability

The code and data of the current research is available at 10.6084/m9.figshare.25712163.
